# Maltreatment and Emotion Recognition Among Brazilian Adolescents

**DOI:** 10.3389/fpsyt.2018.00625

**Published:** 2018-11-26

**Authors:** Guilherme Rodrigues Marta, Victoria Fogaça Doretto, Sandra Scivoletto

**Affiliations:** ^1^Faculdade de Medicina FMUSP, São Paulo Research Foundation (FAPESP), Universidade de São Paulo, São Paulo, Brazil; ^2^Departamento de Psiquiatria, Faculdade de Medicina, Hospital das Clinicas HCFMUSP, Universidade de São Paulo, São Paulo, Brazil; ^3^Psiquiatria da Infância e Adolescencia, Departamento de Psiquiatria, Faculdade de Medicina, Hospital das Clinicas HCFMUSP, Universidade de São Paulo, São Paulo, Brazil

**Keywords:** child abuse, facial expressions, emotion recognition, neglect, maltreatment

## Abstract

The experience of maltreatment can impair child development, including changes in the process of emotions recognition, which may result in impairment of social interactions and behavioral disabilities. In order to measure the association between maltreatment and changes on emotion recognition among Brazilian adolescents, the Emotional Recognition Test on Human Faces (ERTHF) was applied to a sample of 50 adolescents who had suffered different intensities and types of abuse. The social and clinical characteristics of the participants were analyzed and, from ERTHF data, the accuracy and response time for the emotion recognition. Males were 60%, with mean age of 13 years and 3 months; 60% were living in shelters. Emotion recognition changes were associated with intensity and types of maltreatment. Physical neglect (48%) was associated with changes in neutral and negative emotions recognition. Emotional neglect (48%) and emotional abuse (46%) were associated with changes in both positive and negative emotions recognition. Physical abuse (38%) was associated with changes in positive emotion recognition only. False recognition of anger was the most common outcome of maltreatment, being associated with physical neglect (*p* = 0.015) and emotional neglect (*p* = 0.047). Our results point out to the need to add emotional and facial recognition's rehabilitation interventions to better attend the specific demands of maltreated children and to increase the chances of social and family reintegration.

## Introduction

Childhood maltreatment may cause neurocognitive impairments, such as attention, language and decision making, and neuroanatomical changes (1). In addition, maltreatment is associated with several mental disorders: mainly mood disorders, conduct disorder and substance abuse (2), but also anxiety, posttraumatic stress disorder (3, 4), and changes in social behavior (5). The impact of child maltreatment is related to the time and duration of the exposure. Sonuga-Barke et al. (6) compared children institutionalized for less than 6 months with children institutionalized for more than 6 months and found that mental disorders are more frequent in the group with longer institutionalization and tend to persist into adulthood, with emotional changes increasing in adolescence, even after adoption and receiving adequate care for years.

The impact of child maltreatment often transcends the stigma of physical trauma and total lack of care. These experiences can alter the perception of the diverse environments crucial for adequate development, like school, family and work, through the erroneous identification of the expressed emotions, damaging the individuals' social interaction. According to Pollak et al. (7), the ability to recognize emotional expressions allows behavioral adjustment. Maltreated children may associate some facial expressions with traumatic experiences, altering the facial recognition process (8, 9). These children present several difficulties in establishing affective bonds and supportive relationships, leading to the development of behavioral disabilities (10) that generate greater social exclusion and, in the long term, perpetuate the cycle of violence and exclusion (11).

Neglect results from a complex personal, familial and social dynamic (12) and is characterized by chronic absence of adequate care, occurring independently of the financial condition of their parents or caregivers, who fail to meet children's demands. Besides its high prevalence, childhood neglect has been overlooked in scientific research and its impact on child development is generally grouped with other forms of maltreatment (13). Pollak et al. (14) found that physical neglected children had greater difficulty in correctly recognizing facial emotions than children with no history of abuse or even those who had suffered physical abuse. So, negligence would also have a great impact on emotion recognition. Regarding sex differences, Arnsten (3) suggests that estrogen amplifies the response of the prefrontal cortex to environmental aggressions, thus affecting more intensely female individuals. On the other hand, Mouslon et al. (15) verified that girls presented, in general, a superior performance than boys in the emotion recognition.

Previous works on the emotion recognition process have employed a wide range of different research's methodologies, there is also an absence of a standardized and validated instrument to quantify the occurrence of maltreatment and its intensity (14), making it difficult to compare results (16–18). Another important limitation is the lack of control for intellectual quotient (IQ), since this condition can contribute to changes in emotions recognition process (19). It's also highlighted the relatively small samples and lack of analysis of sex differences.

The impact of maltreatment on child development also depends on individual resilience. One of the factors involved in promoting resilience is the environment, which varies across countries due to the particularities of the support system for these victims (20). Therefore, in addition to replicating previous studies, it is necessary to know in depth the impact of childhood maltreatment in the local population in order to develop more effective prevention and treatment strategies.

Our main goal is to study the association between maltreatment and changes in the process of emotion recognition among Brazilian adolescents. We analyzed the association of intensity and different types of maltreatment in the emotion recognition process, considering sex differences and controlling for IQ. We hypothesized that there would be a positive correlation between intensity of maltreatment and impairment in emotion recognition; different types of maltreatment would be associated with different impacts on emotional recognition process, with negligence being associated with greater impact, and males would have more impairment.

## Methods

### Sample

In Brazil, it is estimated that 18,000 children and adolescents suffer daily physical abuse (21). According to Brazil's National Council of Justice there are 47,815 children and adolescents living in shelters in Brazil, with an increase in recent years; 13,674 of those live in São Paulo^1^. For this study, the sample was selected among shelters and vulnerable families that were located close to the Equilibrium Program (22) headquarters. The Equilibrium Program is a community-based multidisciplinary intervention program to assist maltreated and neglected children and adolescents with behavioral and mental problems living in group shelters or with their families but under vulnerable conditions. The first 50 adolescents who fulfilled the inclusion criteria and accepted to participate in the study were included.

Adolescents between 12 and 16 years of age, with history of maltreatment were selected. The age limit was defined based on the minimum age for application of the instruments: the Brazilian version of the CTQ was only validated for those over 12 years old (23). Those with autism spectrum disorder, severe psychiatric or neurological conditions that might compromise adequate understanding of the tests and instruments were excluded. To minimize the possible influence of other variables, were also excluded: left-handed individuals; those with a history of traumatic brain injury, with post-traumatic amnesia greater than 5 min, verified via PTA-Post Traumatic Amnesia scale (24); epilepsy and other neurological disorders; history of brain tumors and; diagnosis of psychoactive substance dependence.

Participants and their legal guardians signed the approved consent form. This study was approved by the Research Ethics Committee of the Hospital das Clinicas HCFMUSP, Faculdade de Medicina, Universidade de Sao Paulo, Brazil (protocol no. 0798/10).

Socio-demographic data, clinical and maltreatment background were obtained through the medical records of the institutions. When necessary, the responsible was called upon to supplement the information. All adolescents underwent a clinical psychiatric evaluation based on the Kiddie-Sads-Present and Lifetime Version (K-SADS-PL) (25). The Brazilian version of the Children's Trauma Questionnaire (CTQ) was applied to identify and rate the severity of emotional abuse and neglect, physical abuse and neglect and sexual abuse (23).

The participants were submitted to the Emotional Recognition Test on Human Faces (ERTHF) (26). The test evaluates separately the skills needed to recognize emotions, such as immediate and late memory of faces and discrimination of emotions, in six distinct steps, one for each skill. In this study, we analyzed the variables obtained in the third step of ERTHF, which evaluates the discrimination of emotions: pictures of different faces and with different facial expressions (anger, fear, sadness, neutral, happy) were shown and the participant was asked to identify distinct types of emotions. The time to answer was also recorded.

### Statistical analysis

Categorical data were described as absolute and relative frequency and numerical variables as mean and standard deviation. Association analyses were performed among socio-demographic characteristics. Correlations among severity of total and each type of maltreatment (emotional neglect, physical neglect, emotional abuse, physical abuse and sexual abuse), socio-demographic and clinical characteristics were calculated with Kruskall-Wallis test and Fischer's exact test. The precision and response time from the emotion recognition test were analyzed according to the different types and severity of maltreatment.

Subsequently, the sample was divided into groups, based on their CTQ scores for maltreatment in general and for each type of maltreatment: comparison group (None or Minimum by CTQ classification) and the group with maltreatment (Low, Moderate and Severe by CTQ classifications) and association analysis were performed with the same variables (presence of total and each type of maltreatment, socio-demographic and clinical characteristics) using Kruskall-Wallis test and Fischer's exact test. Finally, linear regressions were made in which the dependent variable was the correct recognition of the emotion represented in the test and presence of each type of maltreatment.

All analyses were performed using IBM SPSS Statistics for Windows, Version 22.0 (27) and considering a level of significance of 5%.

## Results

### Socio-demographic characteristics

The majority of the participants (60%, *n* = 30) were male; 32% (*n* = 16) were white and 68% (*n* = 34) were black; the mean age of participants was 13 years and 3 months (sd: + 1.415); 90% (*n* = 45) were between 6th and 9th grades and 10% (*n* = 5) in high school. Psychiatric disorders were present in 40% (*n* = 20) and 38% (*n* = 19) used psychotropic medications, of those only 3 used neuroleptics. The most common psychiatric diagnoses were: attention deficit hyperactivity disorder (*n* = 8; 40%) and depressive disorder (*n* = 6; 30%). The majority (60%, *n* = 30) was living in group shelter and 40% (*n* = 20) with family member under high vulnerability conditions; 24% (*n* = 12) of the adolescents lived in extremely poor conditions. The mean IQ was 101.54 (sd: +12.392).

The mean CTQ score was 42.84 (minimum: 25, maximum: 78, sd: +14.401). Following the division of severity of maltreatment proposed by the CTQ, 44% presented none or minimum maltreatment; 30% moderate; 26% severe maltreatment. The most prevalent types of maltreatment were physical and emotional neglect (48%, *n* = 24). The majority (54%, *n* = 27) had suffered more than one type of maltreatment.

### Socio-demographic characteristics and association with types of maltreatment

As shown in Table 1, the only significant associations between socio-demographic characteristics and types of abuse were those living in group shelter had more emotional (α = 9.677, *p* = 0.008; Fisher exact test) and physical neglect (α = 14.419; *p* = 0.001; Fisher exact test) and emotional abuse (α = 6.701; *p* = 0.031; Fisher exact test). The group with moderate physical neglect had lower IQ when compared to the group without physical neglect (*Z* = −3.162; *p* = 0.002; Mann-Whitney test). No association was found between psychiatric disorders or use of psychotropic medications and type of maltreatment.

**Table 1:** Socio-demographic and clinical characteristics and association with types of abuse in 50 adolescents with history of maltreatment.

		**Emotional neglect**			**Physical neglect**			**Emotional abuse**			**Physical abuse**			**Sexual abuse**		
		**None or minimum**	**Moderate**	**Severe**	**None or minimum**	**Moderate**	**Severe**	**None or minimum**	**Moderate**	**Severe**	**None or minimum**	**Moderate**	**Severe**	**None or minimum**	**Moderate**	**Severe**
Sex^*^	Male	16	10	4	15	10	5	18	6	6	19	3	8	28	0	2
	Female	10	4	6	11	3	6	9	3	8	12	1	7	18	2	0
	*α (p)*	2.376 (0.303)			2.534 (0.298)			2.329 (0.329)			0.688 (0.743)			3.509 (0.135)		
Ethnic group^*^	White	11	2	3	12	2	2	10	2	4	10	2	4	15	0	1
	Black	15	12	7	14	11	9	17	7	10	21	2	11	31	2	1
	*α (p)*	3.220 (0.190)			4.621 (0.103)			0.724 (0.778)			0.972 (0.715)			1.213 (1.000)		
Place of residence^*^	Family house	14	6	0	17	2	1	15	3	2	14	2	4	19	0	1
	Group shelter	12	8	10	9	11	10	12	6	12	17	2	11	27	2	1
	*α (p)*	**9.677 (0.008)**			**14.419 (0.001)**			**6.701 (0.031)**			1.717 (0.483)			1.347 (0.767)		
Psychiatric disorders^*^	No	16	9	8	16	6	7	16	4	9	18	2	9	26	2	1
	Yes	10	5	6	10	7	4	11	5	5	13	2	6	20	0	1
	*α (p)*	1.673 (0.481)			1.056 (0.654)			0.967 (0.640)			0.304 (1.000)			1.430 (0.757)		
Psychopharmacological Treatment^*^	No	18	9	4	18	7	6	17	2	9	21	2	8	28	2	1
	Yes	8	5	6	8	6	5	10	4	5	10	2	7	18	0	1
	*α (p)*	2.598 (0.289)			1.308 (0.583)			0.314 (0.929)			1.353 (0.586)			1.283 (0.766)		
Extreme poverty^*^	Não	20	10	8	17	12	9	18	9	11	25	2	11	35	2	1
	Sim	6	4	2	9	1	2	9	0	3	6	2	4	11	0	1
	*α (p)*	0.360 (0.910)			3.409 (0.169)			4.037 (0.115)			2.116 (0.330)			1.455 (0.680)		
Average IQ^**^		102.27	102.36	98.5	105.27	93.77	101.91	103.78	100.89	97.64	103.77	101	97.07	101.09	112.5	101
														
*Z (p)*		0.917 (0.632)			**9.258 (0.010)**			3.716 (0.156)			5.164 (0.076)			0.581 (0.748)		

* *Fisher Test (Monte Carlo significance); significance level of 0.05*.

** *Kruskal-Wallis Test; significance level of 0.05*.

Regarding emotion recognition, there was a significant correlation between the groups with severe maltreatment compared to the group with none or minimum maltreatment and false recognition of angry faces (α = 1.145; *p* = 0.016; Bonferroni significance) and false recognition of happy faces (α = 2.574; *p* = 0.015; Bonferroni significance); between the groups with severe maltreatment compared to the group with moderate maltreatment and false recognition of happy faces (α = 2.323; *p* = 0.035; Bonferroni significance). That is, the greater the intensity of maltreatment, the greater the occurrence of false recognitions of angry and happy faces.

### Response time for emotion recognition

No significant correlations were found between intensity of maltreatment and response time for the recognition of the different emotions evaluated by ERTHF, as shown in Table 2.

**Table 2:** Response time and association with the severity of maltreatment, according to the CTQ score.

		**Anger: correct acknowledgments**	**Anger: false acknowledgments**	**Fear: correct acknowledgments**	**Fear: false acknowledgments**	**Sadness: correct acknowledgments**	**Sadness: false acknowledgments**	**Happiness: correct acknowledgments**	**Happiness: false acknowledgments**	**Neutral: correct acknowledgments**	**Neutral: false acknowledgments**
Participants	Valid^*^	50	29	50	42	50	44	50	35	49	46
	Not attributable^*^	0	21	0	8	0	6	0	15	1	4
Average response time (milisseconds)		2907.52	22.899.828	2742.38	34.701.786	2608.78	3.258.125	2333.08	23.216.571	29.352.714	38.752.543
Standard deviation (milisseconds)		119.373.119	182.306.735	111.998.893	295.808.936	9.645.766	252.629.578	69.508.369	196.030.165	154.317.473	213.436.094
α (p) ^**^		0.209 (0.145)	0.218 (0.255)	0.017 (0.905)	0.250 (0.110)	0.094 (0.514)	0.149 (0.334)	0.191 (0.183)	0.162 (0.353)	−0.205 (0.157)	−0.180 (0.904)

* *In cases where the participant correctly identifies all faces corresponding to an emotion, there is no data to be analyzed in the false recognition fields. For example, there are 21 individuals who did not miss out on recognizing anger, which puts them in the “not attributable” group*.

** *Pearson's test; significance level of 0.05*.

### Association among different types of maltreatment and emotion recognition

**Graph 1:**
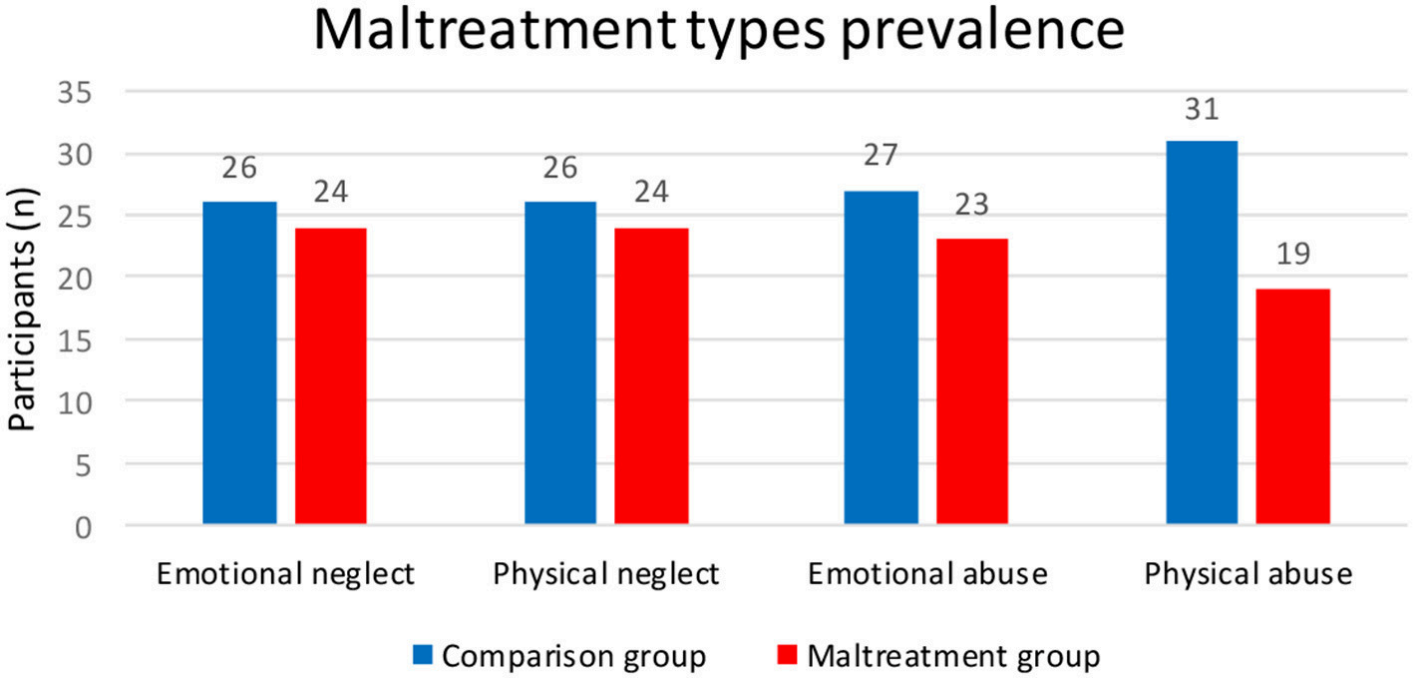
Absolute number of each type of maltreatment in 50 adolescents at risk and vulnerability.

In order to study the impact of different types of maltreatment and precision in the recognition of each type of emotion, the sample was divided into groups: comparison group (none or minimum maltreatment) and the group with each specific type of maltreatment (low to severe maltreatment). As the prevalence of sexual abuse was small (*n* = 4), sexual abuse was not included in the following analysis (Graph 1).

Considering that living in group shelters was significant related to emotional neglect (α = 4.327; *p* = 0.038, Pearson's Chi-square), physical neglect (α = 14.543; *p* = 0.00001; Pearson's Chi-square) and emotional abuse (α = 5.918; *p* = 0.015; Pearson's Chi-square), and that lower IQ was associated with physical neglect (*Z* = −2.605; *p* = 0.009, Mann-Whitney test) and physical abuse (*Z* = −2.201; *p* = 0.028, Mann-Whitney test), the association and linear regression analysis were controlled for these se variables. There was no difference between male and female and recognition of positive and negative emotions.

Table 3 presents the mean ERTHF scores for the comparison and total maltreatment groups. Total maltreatment group showed higher rates of false recognition of anger (α = 2.219; *p* = 0.033; Bonferroni significance).

**Table 3:** Negative and positive emotions identification for the comparison (*n* = 22, 44%) and maltreatment (*n* = 28, 56%) groups.

**Emotion**	**Group**	**Mean acknowledgments (minimum-maximum)**	**Standard deviation**	Correlation^*^
Anger: correct acknowledgments (0–8)	Comparison group	4 (1–8)	1.92725	α = 1.427 (*p* = 0.154)
	Maltreatment group	4.6786 (2–8)	1.36228
Anger: false acknowledgments (0–32)	Comparison group	0.4545 (0–4)	0.9625	α = 2.219 (*p* = 0.033)
	Maltreatment group	1 (0–4)	1.12217
Fear: correct acknowledgments (0–8)	Comparison group	6.7727 (5–8)	0.92231	α = 0.153 (*p* = 0.879)
	Maltreatment group	6.6786	1.46701
Fear: false acknowledgments (0–32)	Comparison group	2.1818 (0–8)	2.12998	α = 0.010 (*p =* 0.992)
	Maltreatment group	2.1429 (0–8)	2.08548
Sadness: correct acknowledgments (0–8)	Comparison group	6.0455 (3–8)	1.43019	α = 0.776 (*p =* 0.438)
	Maltreatment group	5.6071 (1–8)	1.85271
Sadness: false acknowledgments (0–32)	Comparison group	2.5909 (0–9)	2.53845	α = 0.346 (*p =* 0.729)
	Maltreatment group	2.75 (0–10)	2.48886
Happiness: correct acknowledgments (0–8)	Comparison group	7.6818 (6–8)	0.5679	α = 0.739 (*p =* 0.460)
	Maltreatment group	7.3571 (2–8)	1.28277
Happiness: false acknowledgments (0–32)	Comparison group	0.8636 (0–3)	0.94089	α = 0.975 (*p =* 0.329)
	Maltreatment group	1.75 (0–16)	3.08671
Neutral: correct acknowledgments (0–8)	Comparison group	5.9545 (0–8)	2.49718	α = 0.773 (*p =* 0.440)
	Maltreatment group	5.5714 (0–8)	2.45596
Neutral: false acknowledgments (0–32)	Comparison group	3.4545 (0–8)	2.2409	α = 1.540 (*p =* 0.124)
	Maltreatment group	2.4643 (0–6)	1.62121

* *α, Wald chi-square; p, Bonferroni significance*.

Tables 4–7 present the mean ERTHF scores for each type of emotion and for each type of maltreatment. In the test, each emotion is represented by 8 photos, making a total of 40 images; therefore, the maximum values for the correct acknowledgments are 8 and for the false acknowledgments is 32.

**Table 4:** Negative and positive emotions identification for the comparison (*n* = 26, 52%) e emotional abused (*n* = 24, 48%) groups.

**Emotion**	**Group**	**Mean acknowledgments (minimum-maximum)**	**Standard deviation**	Correlation^*^
Anger: correct acknowledgments (0–8)	Comparison group	4.3333 (1–8)	1.98068	α = 0.500 (*p =* 0.799)
	Maltreatment group	4.4348 (2–7)	1.19947
Anger: false acknowledgments (0–32)	Comparison group	0.667 (0–4)	1.07417	α = 0.337 (*p =* 0.280)
	Maltreatment group	0.8696 (0–4)	1.09977
Fear: correct acknowledgments (0–8)	Comparison group	6.9259 (5–8)	0.95780	α = 0.346 (*p =* 0.064)
	Maltreatment group	6.4783 (2–8)	1.50362
Fear: false acknowledgments (0–32)	Comparison group	2.4074 (0–8)	2.35763	α = 0.637 (*p =* 0.342)
	Maltreatment group	1.8696 (0–6)	1.7137
Sadness: correct acknowledgments (0–8)	Comparison group	6.0741 (3–8)	1.4122	α = 0.508 (*p =* 0.190)
	Maltreatment group	5.4783 (1–8)	1.92754
Sadness: false acknowledgments (0–32)	Comparison group	2.8519 (0–9)	2.42905	α = 0.785 (*p =* 0.727)
	Maltreatment group	2.4783 (0–10)	2.59141
Happiness: correct acknowledgments (0–8)	Comparison group	7.6667 (2–8)	1.1767	α = 0.297 (*p =* 0.089)
	Maltreatment group	7.3043 (5–8)	0.82212
Happiness: false acknowledgments (0–32)	Comparison group	0.8889 (0–3)	0.9337	α = 0.560 (*p =* 0.030)
	Maltreatment group	1.9130 (0–16)	3.36983
Neutral: correct acknowledgments (0–8)	Comparison group	5.6296 (0–8)	2.45181	α = 0.753 (*p* = 0.942)
	Maltreatment group	5.8696 (0–8)	2.51006
Neutral: false acknowledgments (0–32)	Comparison group	2.5556 (0–8)	1.84669	α = 0.521 (*p =* 0.028)
	Maltreatment group	3.3043 (0–8)	2.05459

* *α, Wald chi-square; p, Bonferroni significance*.

**Table 5:** Negative and positive emotions identification for the comparison (*n* = 31, 62%) physically abused (*n* = 19, 38%) groups.

**Emotion**	**Group**	**Mean acknowledgments (minimum-maximum)**	**Standard deviation**	Correlation^*^
Anger: correct acknowledgments (0–8)	Comparison group	4.6452 (1–8)	1.81748	α = 0.300 (*p =* 0.884)
	Maltreatment group	3.9474 (1–6)	1.26814
Anger: false acknowledgments (0–32)	Comparison group	0.7097 (0–4)	1.07062	α = 0.300 (*p =* 0.884)
	Maltreatment group	0.8421 (0–4)	1.11869
Fear: correct acknowledgments (0–8)	Comparison group	6.7200 (2–8)	0.92979	α = 0.362 (*p =* 0.718)
	Maltreatment group	2.1600 (0–8)	1.66842
Fear: false acknowledgments (0–32)	Comparison group	2.2258 (0–8)	2.27610	α = 0.599 (*p =* 0.656)
	Maltreatment group	2.0526 (0–6)	1.77869
Sadness: correct acknowledgments (0–8)	Comparison group	5.7742 (2–8)	1.56439	α = 0.480 (*p =* 0.461)
	Maltreatment group	5.8421 (1–8)	1.89336
Sadness: false acknowledgments (0–32)	Comparison group	2.2258 (0–8)	2.61838	α = 0.726 (*p =* 0.995)
	Maltreatment group	2.0526 (0–6)	2.30687
Happiness: correct acknowledgments (0–8)	Comparison group	7.7742 (0–6)	0.49730	α = 0.287 (*p =* 0.011)
	Maltreatment group	7.0526 (2–8)	1.47097
Happiness: false acknowledgments (0–32)	Comparison group	0.8710 (0–3)	0.92166	α = 0.400 (*p =* 0.177)
	Maltreatment group	2.1579 (0–16)	3.65549
Neutral: correct acknowledgments (0–8)	Comparison group	5.7097 (0–8)	2.64819	α = 0.733 (*p* = 0.997)
	Maltreatment group	5.7895 (0–8)	2.17508
Neutral: false acknowledgments (0–32)	Comparison group	3 (0–8)	2.25093	α = 0.574 (*p =* 0.761)
	Maltreatment group	2.7368 (0–6)	1.408

* *α, Wald chi-square; p, Bonferroni significance*.

**Table 6:** Negative and positive emotions identification for the comparison (*n* = 26, 52%) emotionally neglected (*n* = 24, 48%) groups.

**Emotion**	**Group**	**Mean acknowledgments (minimum-maximum)**	**Standard deviation**	Correlation^*^
Anger: correct acknowledgments (0–8)	Comparison group	4.2308 (1–8)	1.7506	α = 0.490 (*p =* 0.776)
	Maltreatment group	4.517 (2–8)	1.5598
Anger: false acknowledgments (0–32)	Comparison group	0.5769 (0–4)	1.02657	α = 3.955 (*p =* 0.047)
	Maltreatment group	0.9583 (0–4)	1.12208
Fear: correct acknowledgments (0–8)	Comparison group	6.6538 (5–8)	0.97744	α = 0.005 (*p =* 0.0946)
	Maltreatment group	6.7919 (2–8)	1.50302
Fear: false acknowledgments (0–32)	Comparison group	2.1923 (0–8)	2.15442	α = 0.006 (*p =* 0.938)
	Maltreatment group	2.1250 (0–8)	2.04966
Sadness: correct acknowledgments (0–8)	Comparison group	6.1154 (4–8)	1.36607	α = 0.497 (*p =* 0.154)
	Maltreatment group	5.4583 (1–8)	1.93321
Sadness: false acknowledgments (0–32)	Comparison group	2.4615 (0–10)	2.4369	α = 1.606 (*p =* 0.205)
	Maltreatment group	2.9167 (0–9)	2.56933
Happiness: correct acknowledgments (0–8)	Comparison group	7.4231 (2–8)	1.23849	α = 0.100 (*p =* 0.752)
	Maltreatment group	7.5833 (5–8)	0.77553
Happiness: false acknowledgments (0–32)	Comparison group	1.0385 (0–3)	1.07632	α = 6.099 (*p =* 0.014)
	Maltreatment group	1.7083 (0–16)	3.30322
Neutral: correct acknowledgments (0–8)	Comparison group	6 (1–8)	2.38328	α = 0.729 (*p* = 0.295)
	Maltreatment group	5.4583 (0–8)	2.55341
Neutral: false acknowledgments (0–32)	Comparison group	3.3077 (0–8)	2.07402	α = 0.574 (*p =* 0.233)
	Maltreatment group	2.4583 (0–6)	1.76879

* *α, Wald chi-square; p, Bonferroni significance*.

**Table 7:** Negative and positive emotions identification for the comparison (*n* = 26, 52%) physically neglected (*n* = 24, 48%) groups.

**Emotion**	**Group**	**Mean acknowledgments (minimum-maximum)**	**Standard deviation**	Correlation^*^
Anger: correct acknowledgments (0–8)	Comparison group	4.1538 (1–8)	1.54123	α = 0.589 (*p =* 0.506)
	Maltreatment group	4.6250 (2–8)	1.76469
Anger: false acknowledgments (0–32)	Comparison group	0.5 (0–4)	0.98995	α = 0.353 (*p =* 0.015)
	Maltreatment group	1.0417 (0–4)	1.12208
Fear: correct acknowledgments (0–8)	Comparison group	7 (5–8)	0.89443	α = 0.359 (*p =* 0.024)
	Maltreatment group	6.4167 (2–8)	1.50121
Fear: false acknowledgments (0–32)	Comparison group	1.9615 (0–5)	1.73161	α = 0.520 (*p =* 0.434)
	Maltreatment group	2.3750 (0–8)	2.42832
Sadness: correct acknowledgments (0–8)	Comparison group	6 (2–8)	1.49666	α = 0.577 (*p =* 0.560)
	Maltreatment group	5.5833 (1–8)	1.86307
Sadness: false acknowledgments (0–32)	Comparison group	2.0385 (0–9)	2.30618	α = 0.816 (*p =* 0.023)
	Maltreatment group	3.3750 (0–10)	2.53347
Happiness: correct acknowledgments (0–8)	Comparison group	7.4615 (2–8)	1.24035	α = 0.353 (*p =* 0.794)
	Maltreatment group	7.5417 (5–8)	0.77903
Happiness: false acknowledgments (0–32)	Comparison group	0.6923 (0–2)	0.83758	α = 0.387 (*p =* 0.098)
	Maltreatment group	2.0833 (0–16)	3.25599
Neutral: correct acknowledgments (0–8)	Comparison group	6.4615 (0–8)	2.23125	α = 0.751 (*p* = 0.001)
	Maltreatment group	4.9583 (0–8)	2.49311
Neutral: false acknowledgments (0–32)	Comparison group	3.7308 (0–8)	2.01112	α = 0.569 (*p =* 0.001)
	Maltreatment group	3.7308 (0–8)	1.47442

* *α, Wald chi-square; p, Bonferroni significance*.

Adolescents victims of physical neglect showed less precision in the recognition of fearful faces (α = 0.359; *p* = 0.024) and of neutral faces (α = 0.751; *p* = 0.001); also showed higher rates of false recognition of angry faces (α = 0.353, *p* = 0.015), false recognition of sad faces (α = 0.816, *p* = 0.023) and lower rate of false recognition of neutral faces (α = 0.569, *p* = 0.001). Those victims of emotional neglect presented more false recognition of angry faces (α = 3.955, *p* = 0.047) and false recognition of happy faces (α = 6.099, *p* = 0.014). Adolescents with a history of emotional abuse had more false recognition of neutral faces (α = 0.521, *p* = 0.028) and more false recognitions of happy faces (α = 0.560, *p* = 0.03). Physical abuse was associated only with less precision in the recognition of happy faces (α = 0.287; *p* = 0.011), without alterations in the recognition of negative or neutral emotions.

## Discussion

To our knowledge, this is the first study to employ the ERTHF with Brazilian adolescents victims of abuse. The results evidenced the association between different types of maltreatment and changes in the recognition process of positive and negative emotions. Neglect, both physical and emotional, had the greatest association with changes on emotion recognition.

We found that the greater the severity of maltreatment, the greater the false recognition of angry faces. Previous studies have showed similar results. Children victims of abuse have lower thresholds for correct recognition of anger, that is, they need a smaller amount of information to accurately recognize anger (8, 28). Ardizzi et al. (29) also found that children living in the streets and victims of abuse recognize faces of anger more accurately compared to other emotions. The lower threshold for anger recognition could be explained as an adaptation and defense mechanism for a repeatedly assaulted organism. Excessive and repeated exposure to anger can cause the individual to recognize this emotion in situations in which it does not exist (e.g., neutral faces). This could lead to socially inadequate reactions, hampering the process of social integration and leading to social rejection. In our study, however, false acknowledgments of anger were associated with emotional and physical neglect, but were not associated with the experience of physical abuse. It is possible that the lower prevalence of physical abuse in our sample (38%), when compared to the prevalence of neglect (48%), prevented the identification of changes in the recognition process of negative emotions. On the other hand, Young et al. (19) did not identify changes in recognition of negative emotions in victims of maltreatment and neglect either. The contradictory results can be explained by methodological differences: Young et al. (19) used the International Affective Picture System (IAPS) (30) and worked with a sample of 547 adults (mean age = 47.1 years; *sd* = 3.44). It is possible that the changes in the emotion recognition associated with the experience of neglect observed in adolescents in our study may be transient and cease to occur in adulthood. The longitudinal monitoring of children and adolescents is crucial to verify the persistence of these changes over time.

For the recognition of positive emotions, we identified higher rates of false recognition of happy faces among adolescent victims of emotional abuse and neglect. With similar results, a previous study showed that children removed from institutional care and allocated to foster care exhibited an attentional bias toward happy faces, that is, they presented the focus of attention preferentially to happy faces (31). On the other hand, Moulson et al. (15) showed that neglected children who lived in institutions needed a greater amount of stimuli to correctly identify cheerful faces. This may be a consequence of the lack of stimulation: without sufficient exposure to joy's expressions, the individual has more difficulty in correctly identifying it. In this way, the positive reinforcement of appropriate behaviors does not occur, which impairs behavior adequacy and social interaction.

Previous studies indicate that victims of maltreatment are more impulsive (32), which could cause errors in the emotions' recognition. Although, there was no correlation between intensity of maltreatment and response time for emotion recognition, which indicates that the false recognitions found in our study cannot be attributed to impulsivity in the responses.

Psychiatric disorders tend to impair emotion recognition. Kohler et al. (33) worked with a population of 89 participants, of whom 28 had a diagnosis of schizophrenia; in the study, which also used the ERTHF as an instrument, schizophrenic patients presented a lower performance than the control group, mainly in relation to the neutral and fear faces recognition, not associated with the use of antipsychotics medications. Other psychiatric diagnoses, such as depression, substance abuse, and bipolar affective disorder are also associated with errors in the recognition of emotions, requiring demonstrated emotions of greater intensity to be correctly recognized. In our study, we found no association between changes in the recognition of emotions and presence of psychiatric diagnoses or use of psychotropic drugs.

Neglect was the type of maltreatment with the greatest impact on emotions' recognition, which is consistent with previous studies (14, 34). Physical and emotional neglect were associated with changes in the recognition of all types of emotions. Physical neglect caused false acknowledgments of angry, sad and neutral faces, that is, individuals identified neutral faces on faces that expressed emotions and vice versa. Physical neglect was also associated with impairments on fear recognition. On the other hand, emotional neglect was associated with false acknowledgments of angry and happy faces. Similarly, Young et al. (19) accompanied abused children compared to children without this history; when in early adulthood, those with a history of maltreatment, particularly neglect and sexual abuse, had a deficit in the recognition of positive emotions, but not in negative emotions. In our study, it was not possible to verify the impact of sexual abuse on the recognition of emotions since the low prevalence of sexual abuse in our sample (*n* = 4). However, the joint analysis of previous studies and our results shows that neglect is associated with changes not only in positive emotions, but also in negative and neutral ones, with impact already in childhood and persistent consequences throughout the development of the individual, as presented by Young et al. (19).

Contrary to our initial hypothesis, no sex difference was found in both positive and negative emotion recognition in our study, as well as the work of Masten et al. (9) and Pollak et al. (14).

This study presents some limitations regarding the sample and the methodology used. Although our sample size was similar to other studies investigating multiple emotions, other significant associations could possibly be found if the sample was larger. In fact, it is a small sample and limited to few shelters and families located in Sao Paulo – Brazil's greatest city. In this way, the results cannot be generalized to other Brazilian regions or even other countries. It would be also possible to test the influence of other variables in the emotion recognition process in a larger sample, such as psychiatric disorders -specially the presence of light symptoms of autism spectrum disorder-, use of psychotropic medications and history of sexual abuse, once its presence may significantly impact the recognition of facial emotions. Future studies should adopt a standardized instrument for the investigation of mild symptoms of autism spectrum disorder. Nevertheless, even with a small sample, significant results were found and may contribute to the existing literature.

Another important aspect is the absence of a control group with individuals with no maltreatment experience, which would allow a more accurate analysis of the impact of each type of abuse. Obtaining control groups with no history of maltreatment would imply in a very different development context from the sample, which would also add other confounding variables to the analyses. Thus, we opted to adopt analyses with comparison groups, with individuals without the experience of a specific type of maltreatment, but who experienced other types according to the CTQ definitions (23). The division of groups with and without specific subtypes of maltreatment experiences was based on the criteria established by the instrument of measurement of maltreatment adopted, the CTQ, which is an instrument used in several other studies but that can bring bias in the groups' division. Future research could apply more than one instrument to track and measure maltreatment to ensure better characterization of maltreatment experiences and data reliability. However, chronic and intrauterine polivitimization are characteristics of the studied population (35) and this study aimed to analyze the impact of maltreatment as close as possible to the reality to which this population is exposed: chronic polivitimization.

Additionally, it was not possible to precisely identify the timing of the onset and the duration of the maltreatment. The majority of adolescents were institutionalized due to dysfunctional family environments and many families were unable to provide precise information, either due to judicial impediment or due to the presence of psychiatric difficulties that prevented access to reliable information (36). Cross-sectional studies allow identification of association, but not cause-effect establishment. Adolescents who presented changes in emotion recognition could have presented these difficulties early in life, which could lead to poor caregivers' investments (neglect) or even generate situations of abuse. Longitudinal studies are needed to specifically address this aspect.

Regarding the methodology used, the ERTHF present photos of faces with different emotions, which is a representation that is not trustworthy to reality. Future studies could complete the assessment through tests that require the situational understanding in which emotions occur. Neuroimaging studies, especially functional, would be important in identifying brain circuits involved in tasks of emotions recognition and the impact of maltreatment on neurocognitive and emotional functioning. Another methodological point to be considered is the possible retrospective falsification bias and recall bias in reporting maltreatment; however, this must be considered in all retrospective studies with this type of population.

The objective of this study was to evaluate the possible impact of maltreatment experiences in the process of emotion recognition. As our results pointed out, it does have an impact in the emotion recognition process. Future studies may now evaluate the impact of these alterations on the quality of life of these individuals. Other life experiences may have a compensatory effect and lessen the possible negative impact on the final quality of life, but research is needed that employ objective instruments to measure quality of life.

In order to mitigate the impact of maltreatment on child development, it is important to promote resilience and local environment factors are particularly important, as pointed by Ungar (20). Despite the limitations pointed out, this is the first study to analyze the association between maltreatment and impact on the emotions recognition process among Brazilian adolescents with a cross-sectional methodology. Future studies should focus on the longitudinal follow-up of the participants in order to evaluate the persistence of these changes and to identify protective factors. In the treatment of this population, specific interventions are necessary to improve emotion recognition to promote social abilities and thus improving social interaction.

## Conclusion

Properly recognizing facial emotions is a key factor involved in the overall process of emotional perception, which is fundamental for establishing healthy relationships and for the overall promotion of health and good quality of life. Both intensity and types of maltreatment, especially neglect, are associated with significant impact on emotions' recognition, either by increased emotional response (false recognition) or by a decrease in correct identification (recognition error). Therefore, our results point out to the need to add emotional and facial recognition's rehabilitation interventions to better attend the specific demands of maltreated children and to increase the chances of social and family reintegration.

## Author contributions

GM is the main author and SS, his advisor, as part of a scientific initiation project. GM and SS contributed conception and design of the study. SS participated actively in all phases of the project, encouraging GM to widen his vision and knowledge about this relevant theme. GM organized the database, performed the statistical analysis and wrote the first draft of the manuscript. SS and VD wrote sections of the manuscript. All authors contributed to manuscript revision, read and approved the submitted version.

### Conflict of interest statement

The authors declare that the research was conducted in the absence of any commercial or financial relationships that could be construed as a potential conflict of interest.

## Footnotes

^1^http://www.cnj.jus.br/cnca/relatorio/ControleRelatorio.php?action=acolhido (Accessed October 22).

## References

[1] Oliveira PAd Scivoletto S Cunha PJ Estudos neuropsicológicos e de neuroimagem associados ao estresse emocional na infância e adolescência. Rev de Psiquiatria Clín. (2010) 37:271–9. 10.1590/S0101-60832010000600004

[2] Scomparini LB Dos Santos B Rosenheck RA Scivoletto S. Association of child maltreatment and psychiatric diagnosis in Brazilian children and adolescents. Clinics (2013) 68:1096–102. 10.6061/clinics/2013(08)06 24037004 PMC3757160

[3] Arnsten AF. Stress signalling pathways that impair prefrontal cortex structure and function. Nat Rev Neurosci. (2009) 10:410–22. 10.1038/nrn2648 19455173 PMC2907136

[4] Öztürk A Kiliç A Deveci E Kirpinar I. Investigation of facial emotion recognition, alexithymia, and levels of anxiety and depression in patients with somatic symptoms and related disorders. Neuropsychiatr Dis Treat. (2016) 12:1047–53. 10.2147/NDT.S106989 27199559 PMC4857827

[5] Ometto M de Oliveira PA Milioni AL Dos Santos B Scivoletto S Busatto GF. Social skills and psychopathic traits in maltreated adolescents. Eur Child Adoles Psychiatry (2016) 25:397–405. 10.1007/s00787-015-0744-y 26224584

[6] Sonuga-Barke EJ Kennedy M Kumsta R Knights N Golm D Rutter M. Child-to-adult neurodevelopmental and mental health trajectories after early life deprivation: the young adult follow-up of the longitudinal English and Romanian Adoptees study. Lancet (2017) 389:1539–48. 10.1016/S0140-6736(17)30045-4 28237264

[7] Pollak SD Messner M Kistler DJ Cohn JF. Development of perceptual expertise in emotion recognition. Cognition (2009) 110:242–7. 10.1016/j.cognition.2008.10.010 19059585 PMC2673797

[8] Pollak SD Sinha P. Effects of early experience on children's recognition of facial displays of emotion. Dev Psychol. (2002) 38:784–91. 10.1037/0012-1649.38.5.784 12220055

[9] Masten CL Guyer AE Hodgdon HB McClure EB Charney DS Ernst M. Recognition of facial emotions among maltreated children with high rates of post-traumatic stress disorder. Child Abuse Neglect (2008) 32:139–53. 10.1016/j.chiabu.2007.09.006 18155144 PMC2268025

[10] Humphreys KL Nelson CA Fox NA Zeanah CH. Signs of reactive attachment disorder and disinhibited social engagement disorder at age 12 years: Effects of institutional care history and high-quality foster care. Dev Psychopathol. (2017) 29:675–84. 10.1017/S0954579417000256 28401844 PMC5777580

[11] Stefanovics EA Mauro Filho V Rosenheck RA Scivoletto S. Functional outcomes of maltreated children and adolescents in a community-based rehabilitation program in Brazil: Six-month improvement and baseline predictors. Child Abuse Neglect (2014) 38:1231–7. 10.1016/j.chiabu.2013.10.025 24300697

[12] Leadbeater B Dodgen D Solarz A The resilience revolution: a paradigm shift for research and policy? In: Peters RD Leadbeater B McMahon RJ editors. Resilience in Children, Families, and Communities: Linking Context to Practice and Policy. New York, NY: Kluwer Academic; Plenum Publishers (2005). p. 47–61. 10.1007/0-387-23824-7_4

[13] Mersky JP Topitzes J. Comparing early adult outcomes of maltreated and non-maltreated children: a prospective longitudinal investigation. Child Youth Serv Rev. (2010) 32:1086–96. 10.1016/j.childyouth.2009.10.018 27667886 PMC5034869

[14] Pollak SD Cicchetti D Hornung K Reed A. Recognizing emotion in faces: developmental effects of child abuse and neglect. Dev Psychol. (2000) 36:679–688. 10.1037/0012-1649.36.5.679 10976606

[15] Moulson MC Shutts K Fox NA Zeanah CH Spelke ES Nelson CA. Effects of early institutionalization on the development of emotion processing: a case for relative sparing? Dev Sci. (2015) 18:298–313. 10.1111/desc.12217 25039290 PMC4297604

[16] Morgan JK Izard CE King KA. Construct validity of the emotion matching task: preliminary evidence for convergent and criterion validity of a new emotion knowledge measure for young children. Soc Dev. (2010) 19:52–70. 10.1111/j.1467-9507.2008.00529.x 20376197 PMC2850106

[17] Gao X Maurer D. Influence of intensity on children's sensitivity to happy, sad, and fearful facial expressions. J Exp Child Psychol. (2009) 102:503–21. 10.1016/j.jecp.2008.11.002 19124135

[18] Izard C Fine S Schultz D Mostow A Ackerman B Youngstrom E. Emotion knowledge as a predictor of social behavior and academic competence in children at risk. Psychol Sci. (2001) 12:18–23. 10.1111/1467-9280.00304 11294223

[19] Young JC Widom CS. Long-term effects of child abuse and neglect on emotion processing in adulthood. Child Abuse Neglect (2014) 38:1369–81. 10.1016/j.chiabu.2014.03.008 24747007 PMC4117717

[20] Ungar M. Resilience after maltreatment: the importance of social services as facilitators of positive adaptation. Child Abuse Neglect (2013) 37:110–5. 10.1016/j.chiabu.2012.08.004 23260114

[21] Oliveira VLAd Ribeiro CR Albuquerque MCd Notificação obrigatória da violência ou suspeita de violência contra crianças e adolescentes: construindo uma rede de proteção. Divulg saúde debate (2003) 66–72.

[22] Marques AH Oliveira PA Scomparini LB Silva AC Doretto V Medeiros Filho MVd. Community-based Global Health Program for maltreated children and adolescents in Brazil: the Equilibrium Program. Front Psychiatry (2015) 6:102. 10.3389/fpsyt.2015.00102 26283972 PMC4519654

[23] Grassi-Oliveira R Stein LM Pezzi JC. Translation and content validation of the Childhood Trauma Questionnaire into Portuguese language. Rev Saude Publica (2006) 40:249–55. 10.1590/S0034-89102006000200010 16583035

[24] Shores EA Marosszeky J Sandanam J Batchelor J. Preliminary validation of a clinical scale for measuring the duration of post-traumatic amnesia. Med J Aust. (1986) 144:569–72. 3713586 10.5694/j.1326-5377.1986.tb112311.x

[25] Kaufman J Birmaher B Brent D Rao U Flynn C Moreci P. Schedule for affective disorders and schizophrenia for school-age children-present and lifetime version (K-SADS-PL): initial reliability and validity data. J Am Acad Child Adolesc Psychiatry (1997) 36:980–8. 10.1097/00004583-199707000-00021 9204677

[26] Gur RC Ragland JD Moberg PJ Turner TH Bilker WB Kohler C. Computerized neurocognitive scanning:: I. Methodology and validation in healthy people. Neuropsychopharmacology (2001) 25:766–76. 10.1016/S0893-133X(01)00278-0 11682260

[27] IBM Corp N IBM SPSS Statistics for Windows. Version 22 (2013).

[28] Sullivan MW Bennett DS Carpenter K Lewis M. Emotion knowledge in young neglected children. Child Maltreat. (2008) 13:301–6. 10.1177/1077559507313725 18299632 PMC3772536

[29] Ardizzi M Martini F Umiltà MA Evangelista V Ravera R Gallese V. Impact of childhood maltreatment on the recognition of facial expressions of emotions. PLoS ONE (2015) 10:e0141732. 10.1371/journal.pone.0141732 26509890 PMC4624998

[30] Lang PJ Bradley MM Cuthbert BN International affective picture system (IAPS): Technical manual and affective ratings. NIMH Center Study Emotion Attention (1997):39–58.

[31] Troller-Renfree S McDermott JM Nelson CA Zeanah CH Fox NA. The effects of early foster care intervention on attention biases in previously institutionalized children in Romania. Dev Sci. (2015) 18:713–22. 10.1111/desc.12261 25439678 PMC4447605

[32] Minzenberg MJ Poole JH Vinogradov S. Adult social attachment disturbance is related to childhood maltreatment and current symptoms in borderline personality disorder. J Nervous Mental Dis. (2006) 194:341–8. 10.1097/01.nmd.0000218341.54333.4e 16699383

[33] Kohler CG Turner TH Bilker WB Brensinger CM Siegel SJ Kanes SJ. Facial emotion recognition in schizophrenia: intensity effects and error pattern. Am J Psychiatry (2003) 160:1768–74. 10.1176/appi.ajp.160.10.1768 14514489

[34] Doretto V Scivoletto S. Effects of Early neglect experience on recognition and processing of facial expressions: a systematic review. Brain Sci. (2018) 8:10. 10.3390/brainsci8010010 29316648 PMC5789341

[35] Finkelhor D Ormrod RK Turner HA. Poly-victimization: a neglected component in child victimization. Child Abuse Neglect (2007) 31:7–26. 10.1016/j.chiabu.2006.06.008 17224181

[36] Scivoletto S da Silva TF Rosenheck RA. Child psychiatry takes to the streets: a developmental partnership between a university institute and children and adolescents from the streets of Sao Paulo, Brazil. Child Abuse Neglect (2011) 35:89–95. 10.1016/j.chiabu.2010.11.003 21377731

